# Our Sunshine

**Published:** 1857-08

**Authors:** 


### OUR SUNSHINE.

A WORTHY dominie, whose heart flows over with lovingness towards all his kind, writes, "I send my subscription to keep the machinery in operation for 1857. Ever since I got a little of your sunshine, I have desired its continuance. Whether in *propria personæ*, or through the medium of the press, it always does me good. I hope ere long to hold the veritable luminary by the hand, and would be glad to see your face in my study, as of yore."

